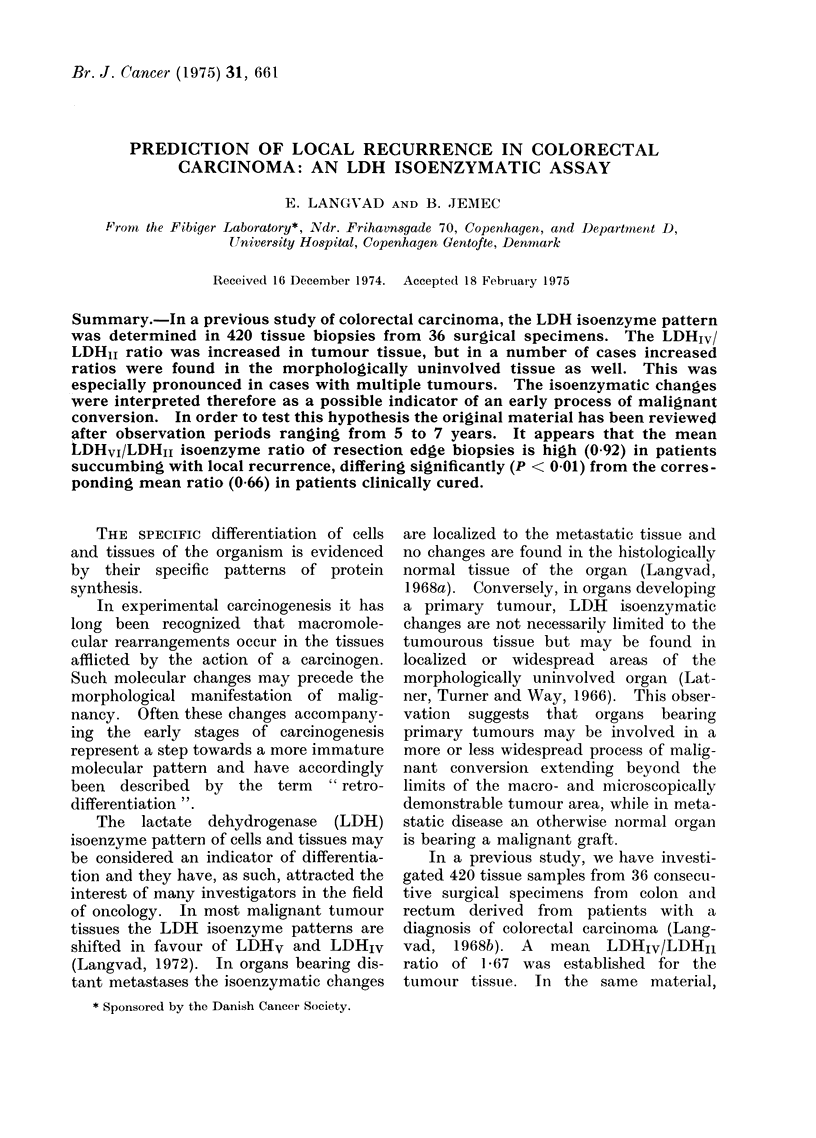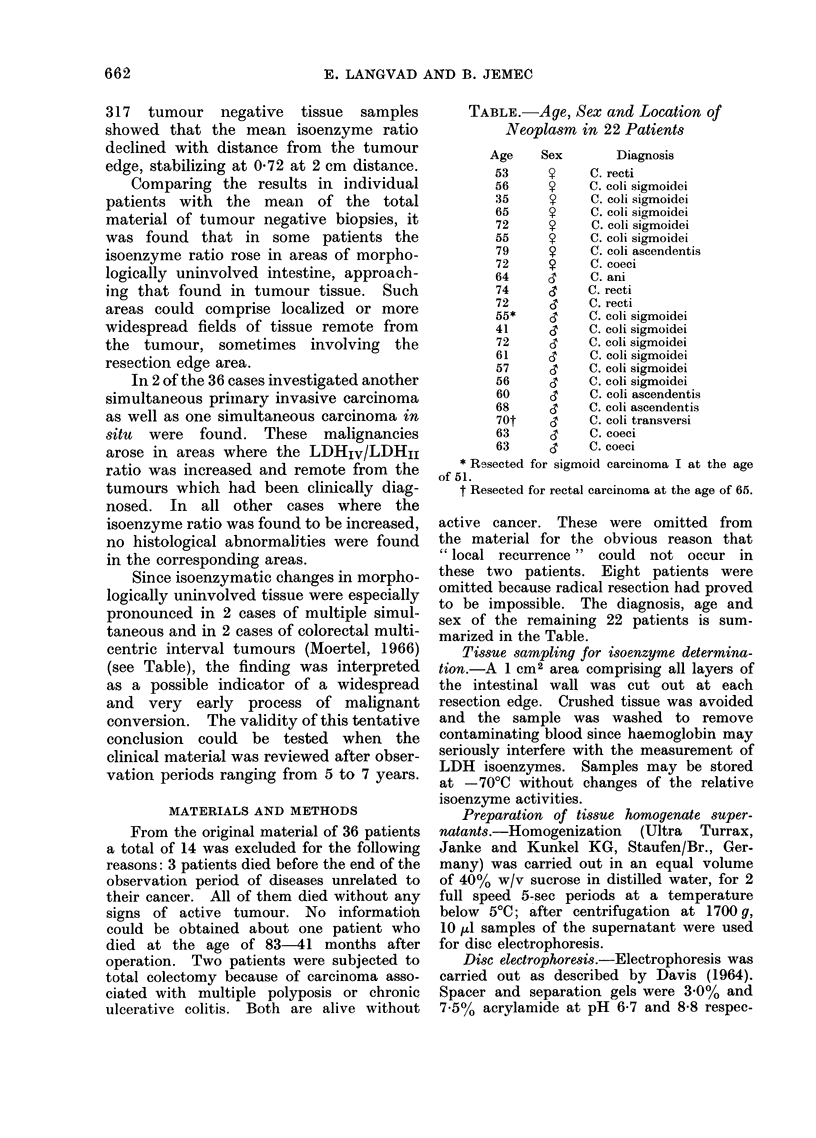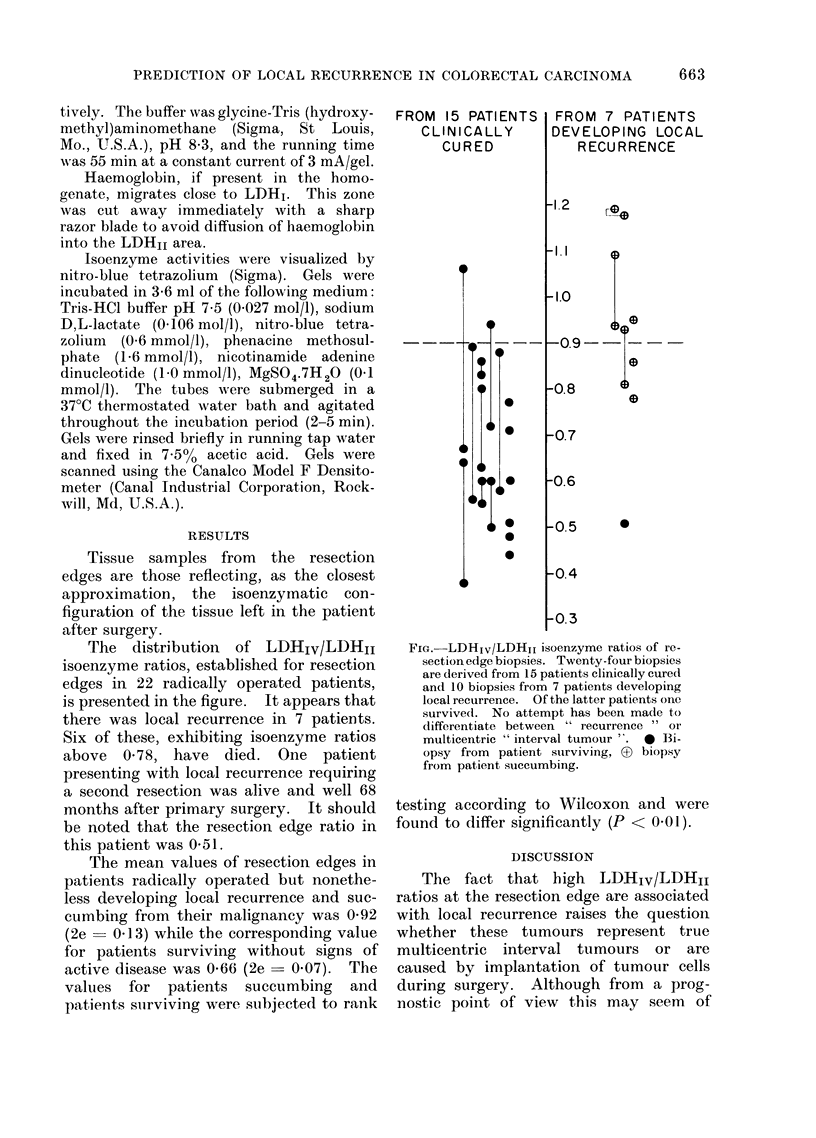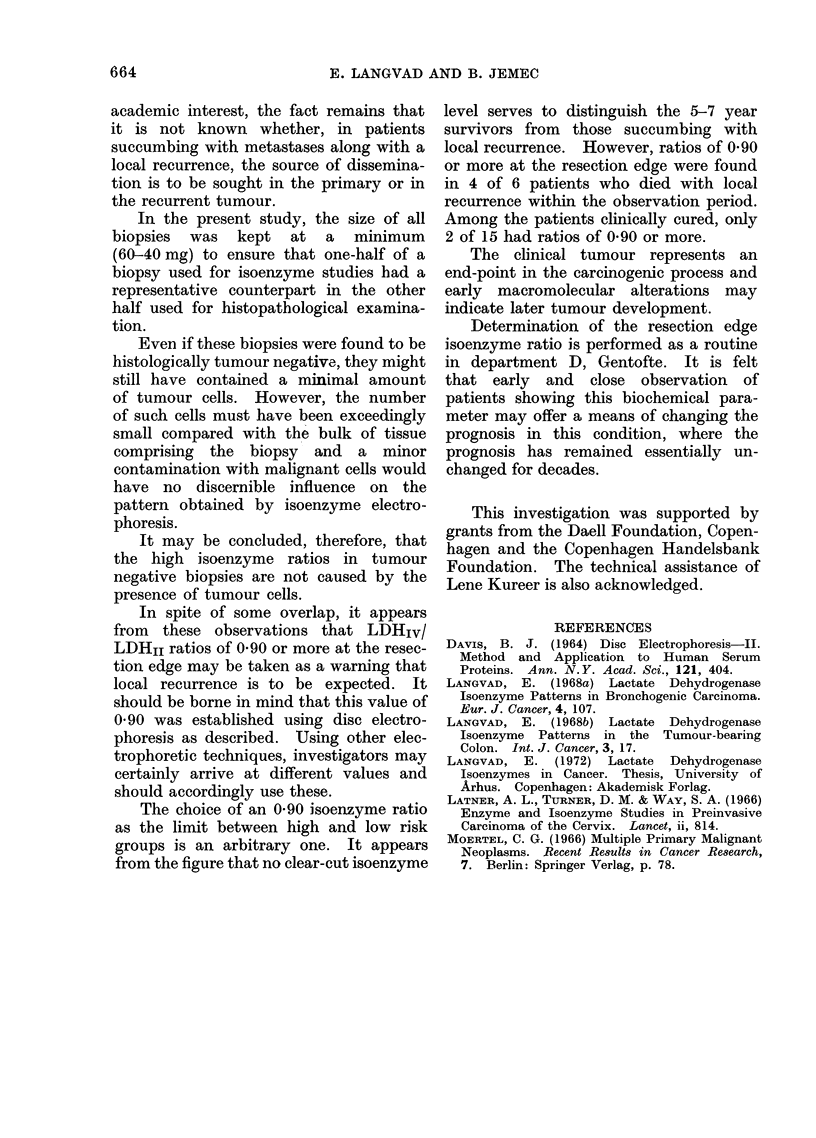# Prediction of local recurrence in colorectal carcinoma: an LDH isoenzymatic assay.

**DOI:** 10.1038/bjc.1975.113

**Published:** 1975-06

**Authors:** E. Langvad, B. Jemec

## Abstract

In a previous study of colorectal carcinoma, the LDH isoenzyme pattern was determined in 420 tissue biopsies from 36 surgical specimens. The LDHIV/LDHII ratio was increased in tumour tissue, but in a number of cases increased ratios were found in the morphologically uninvolved tissue as well. This was especially pronounced in cases with multiple tumours. The isoenzymatic changes were interpreted therefore as a possible indicator of an early process of malignant conversion. In order to test this hypothesis the original material has been reviewed after observation periods ranging from 5 to 7 years. It appears that the mean LDHVI/LDHII isoenzyme ratio of resection edge biopsies is high (0-92) in patients succumbing with local recurrence, differing significantly (P less than 0-01) from the corresponding mean ratio (0-66) in patients clinically cured.


*Br. J. Cancer* (1975) **31**, 661

### PREDICTION OF LOCAL RECURRENCE IN COLORECTAL CARCINOMA: AN LDH ISOENZYMATIC ASSAY

E. LANGVAD AND B. JEMEC

*From the Fibiger Laboratory*, Ndr. Frihavnsgade 70, Copenhagen, and Department D, University Hospital, Copenhagen Gentofte, Denmark*

Received 16 December 1974. Accepted 18 February 1975

**Summary.—In a previous study of colorectal carcinoma, the LDH isoenzyme pattern was determined in 420 tissue biopsies from 36 surgical specimens. The $LDH_{IV}$/$LDH_{II}$ ratio was increased in tumour tissue, but in a number of cases increased ratios were found in the morphologically uninvolved tissue as well. This was especially pronounced in cases with multiple tumours. The isoenzymatic changes were interpreted therefore as a possible indicator of an early process of malignant conversion. In order to test this hypothesis the original material has been reviewed after observation periods ranging from 5 to 7 years. It appears that the mean $LDH_{VI}$/$LDH_{II}$ isoenzyme ratio of resection edge biopsies is high (0·92) in patients succumbing with local recurrence, differing significantly ($P < 0{\cdot}01$) from the corresponding mean ratio (0·66) in patients clinically cured.**

THE SPECIFIC differentiation of cells and tissues of the organism is evidenced by their specific patterns of protein synthesis.

In experimental carcinogenesis it has long been recognized that macromolecular rearrangements occur in the tissues afflicted by the action of a carcinogen. Such molecular changes may precede the morphological manifestation of malignancy. Often these changes accompanying the early stages of carcinogenesis represent a step towards a more immature molecular pattern and have accordingly been described by the term "retro-differentiation".

The lactate dehydrogenase (LDH) isoenzyme pattern of cells and tissues may be considered an indicator of differentiation and they have, as such, attracted the interest of many investigators in the field of oncology. In most malignant tumour tissues the LDH isoenzyme patterns are shifted in favour of $LDH_V$ and $LDH_{IV}$ (Langvad, 1972). In organs bearing distant metastases the isoenzymatic changes are localized to the metastatic tissue and no changes are found in the histologically normal tissue of the organ (Langvad, 1968*a*). Conversely, in organs developing a primary tumour, LDH isoenzymatic changes are not necessarily limited to the tumourous tissue but may be found in localized or widespread areas of the morphologically uninvolved organ (Latner, Turner and Way, 1966). This observation suggests that organs bearing primary tumours may be involved in a more or less widespread process of malignant conversion extending beyond the limits of the macro- and microscopically demonstrable tumour area, while in metastatic disease an otherwise normal organ is bearing a malignant graft.

In a previous study, we have investigated 420 tissue samples from 36 consecutive surgical specimens from colon and rectum derived from patients with a diagnosis of colorectal carcinoma (Langvad, 1968*b*). A mean $LDH_{IV}$/$LDH_{II}$ ratio of 1·67 was established for the tumour tissue. In the same material,

* Sponsored by the Danish Cancer Society.

317 tumour negative tissue samples showed that the mean isoenzyme ratio declined with distance from the tumour edge, stabilizing at 0·72 at 2 cm distance.

Comparing the results in individual patients with the mean of the total material of tumour negative biopsies, it was found that in some patients the isoenzyme ratio rose in areas of morphologically uninvolved intestine, approaching that found in tumour tissue. Such areas could comprise localized or more widespread fields of tissue remote from the tumour, sometimes involving the resection edge area.

In 2 of the 36 cases investigated another simultaneous primary invasive carcinoma as well as one simultaneous carcinoma *in situ* were found. These malignancies arose in areas where the $LDH_{IV}/LDH_{II}$ ratio was increased and remote from the tumours which had been clinically diagnosed. In all other cases where the isoenzyme ratio was found to be increased, no histological abnormalities were found in the corresponding areas.

Since isoenzymatic changes in morphologically uninvolved tissue were especially pronounced in 2 cases of multiple simultaneous and in 2 cases of colorectal multicentric interval tumours (Moertel, 1966) (see Table), the finding was interpreted as a possible indicator of a widespread and very early process of malignant conversion. The validity of this tentative conclusion could be tested when the clinical material was reviewed after observation periods ranging from 5 to 7 years.

### MATERIALS AND METHODS

From the original material of 36 patients a total of 14 was excluded for the following reasons: 3 patients died before the end of the observation period of diseases unrelated to their cancer. All of them died without any signs of active tumour. No information could be obtained about one patient who died at the age of 83—41 months after operation. Two patients were subjected to total colectomy because of carcinoma associated with multiple polyposis or chronic ulcerative colitis. Both are alive without active cancer. These were omitted from the material for the obvious reason that "local recurrence" could not occur in these two patients. Eight patients were omitted because radical resection had proved to be impossible. The diagnosis, age and sex of the remaining 22 patients is summarized in the Table.

TABLE.—*Age, Sex and Location of Neoplasm in 22 Patients*

| Age | Sex | Diagnosis |
|---|---|---|
| 53 | ♀ | C. recti |
| 56 | ♀ | C. coli sigmoidei |
| 35 | ♀ | C. coli sigmoidei |
| 65 | ♀ | C. coli sigmoidei |
| 72 | ♀ | C. coli sigmoidei |
| 55 | ♀ | C. coli sigmoidei |
| 79 | ♀ | C. coli ascendentis |
| 72 | ♀ | C. coeci |
| 64 | ♂ | C. ani |
| 74 | ♂ | C. recti |
| 72 | ♂ | C. recti |
| 55* | ♂ | C. coli sigmoidei |
| 41 | ♂ | C. coli sigmoidei |
| 72 | ♂ | C. coli sigmoidei |
| 61 | ♂ | C. coli sigmoidei |
| 57 | ♂ | C. coli sigmoidei |
| 56 | ♂ | C. coli sigmoidei |
| 60 | ♂ | C. coli ascendentis |
| 68 | ♂ | C. coli ascendentis |
| 70† | ♂ | C. coli transversi |
| 63 | ♂ | C. coeci |
| 63 | ♂ | C. coeci |

* Resected for sigmoid carcinoma I at the age of 51.

† Resected for rectal carcinoma at the age of 65.

*Tissue sampling for isoenzyme determination.*—A 1 $cm^2$ area comprising all layers of the intestinal wall was cut out at each resection edge. Crushed tissue was avoided and the sample was washed to remove contaminating blood since haemoglobin may seriously interfere with the measurement of LDH isoenzymes. Samples may be stored at −70°C without changes of the relative isoenzyme activities.

*Preparation of tissue homogenate supernatants.*—Homogenization (Ultra Turrax, Janke and Kunkel KG, Staufen/Br., Germany) was carried out in an equal volume of 40% w/v sucrose in distilled water, for 2 full speed 5-sec periods at a temperature below 5°C; after centrifugation at 1700 *g*, 10 μl samples of the supernatant were used for disc electrophoresis.

*Disc electrophoresis.*—Electrophoresis was carried out as described by Davis (1964). Spacer and separation gels were 3·0% and 7·5% acrylamide at pH 6·7 and 8·8 respectively. The buffer was glycine-Tris (hydroxymethyl)aminomethane (Sigma, St Louis, Mo., U.S.A.), pH 8·3, and the running time was 55 min at a constant current of 3 mA/gel.

Haemoglobin, if present in the homogenate, migrates close to $LDH_I$. This zone was cut away immediately with a sharp razor blade to avoid diffusion of haemoglobin into the $LDH_{II}$ area.

Isoenzyme activities were visualized by nitro-blue tetrazolium (Sigma). Gels were incubated in 3·6 ml of the following medium: Tris-HCl buffer pH 7·5 (0·027 mol/l), sodium D,L-lactate (0·106 mol/l), nitro-blue tetrazolium (0·6 mmol/l), phenacine methosulphate (1·6 mmol/l), nicotinamide adenine dinucleotide (1·0 mmol/l), $MgSO_4.7H_2O$ (0·1 mmol/l). The tubes were submerged in a 37°C thermostated water bath and agitated throughout the incubation period (2–5 min). Gels were rinsed briefly in running tap water and fixed in 7·5% acetic acid. Gels were scanned using the Canalco Model F Densitometer (Canal Industrial Corporation, Rockwill, Md, U.S.A.).

### RESULTS

Tissue samples from the resection edges are those reflecting, as the closest approximation, the isoenzymatic configuration of the tissue left in the patient after surgery.

The distribution of $LDH_{IV}/LDH_{II}$ isoenzyme ratios, established for resection edges in 22 radically operated patients, is presented in the figure. It appears that there was local recurrence in 7 patients. Six of these, exhibiting isoenzyme ratios above 0·78, have died. One patient presenting with local recurrence requiring a second resection was alive and well 68 months after primary surgery. It should be noted that the resection edge ratio in this patient was 0·51.

The mean values of resection edges in patients radically operated but nonetheless developing local recurrence and succumbing from their malignancy was 0·92 (2e = 0·13) while the corresponding value for patients surviving without signs of active disease was 0·66 (2e = 0·07). The values for patients succumbing and patients surviving were subjected to rank

FIG.—$LDH_{IV}/LDH_{II}$ isoenzyme ratios of resection edge biopsies. Twenty-four biopsies are derived from 15 patients clinically cured and 10 biopsies from 7 patients developing local recurrence. Of the latter patients one survived. No attempt has been made to differentiate between "recurrence" or multicentric "interval tumour". ● Biopsy from patient surviving, ⊕ biopsy from patient succumbing.

testing according to Wilcoxon and were found to differ significantly ($P < 0{\cdot}01$).

### DISCUSSION

The fact that high $LDH_{IV}/LDH_{II}$ ratios at the resection edge are associated with local recurrence raises the question whether these tumours represent true multicentric interval tumours or are caused by implantation of tumour cells during surgery. Although from a prognostic point of view this may seem of academic interest, the fact remains that it is not known whether, in patients succumbing with metastases along with a local recurrence, the source of dissemination is to be sought in the primary or in the recurrent tumour.

In the present study, the size of all biopsies was kept at a minimum (60–40 mg) to ensure that one-half of a biopsy used for isoenzyme studies had a representative counterpart in the other half used for histopathological examination.

Even if these biopsies were found to be histologically tumour negative, they might still have contained a minimal amount of tumour cells. However, the number of such cells must have been exceedingly small compared with the bulk of tissue comprising the biopsy and a minor contamination with malignant cells would have no discernible influence on the pattern obtained by isoenzyme electrophoresis.

It may be concluded, therefore, that the high isoenzyme ratios in tumour negative biopsies are not caused by the presence of tumour cells.

In spite of some overlap, it appears from these observations that $LDH_{IV}/LDH_{II}$ ratios of 0·90 or more at the resection edge may be taken as a warning that local recurrence is to be expected. It should be borne in mind that this value of 0·90 was established using disc electrophoresis as described. Using other electrophoretic techniques, investigators may certainly arrive at different values and should accordingly use these.

The choice of an 0·90 isoenzyme ratio as the limit between high and low risk groups is an arbitrary one. It appears from the figure that no clear-cut isoenzyme level serves to distinguish the 5–7 year survivors from those succumbing with local recurrence. However, ratios of 0·90 or more at the resection edge were found in 4 of 6 patients who died with local recurrence within the observation period. Among the patients clinically cured, only 2 of 15 had ratios of 0·90 or more.

The clinical tumour represents an end-point in the carcinogenic process and early macromolecular alterations may indicate later tumour development.

Determination of the resection edge isoenzyme ratio is performed as a routine in department D, Gentofte. It is felt that early and close observation of patients showing this biochemical parameter may offer a means of changing the prognosis in this condition, where the prognosis has remained essentially unchanged for decades.

This investigation was supported by grants from the Daell Foundation, Copenhagen and the Copenhagen Handelsbank Foundation. The technical assistance of Lene Kureer is also acknowledged.

### REFERENCES

Davis, B. J. (1964) Disc Electrophoresis—II. Method and Application to Human Serum Proteins. *Ann. N.Y. Acad. Sci.*, **121**, 404.

Langvad, E. (1968*a*) Lactate Dehydrogenase Isoenzyme Patterns in Bronchogenic Carcinoma. *Eur. J. Cancer*, **4**, 107.

Langvad, E. (1968*b*) Lactate Dehydrogenase Isoenzyme Patterns in the Tumour-bearing Colon. *Int. J. Cancer*, **3**, 17.

Langvad, E. (1972) Lactate Dehydrogenase Isoenzymes in Cancer. Thesis, University of Århus. Copenhagen: Akademisk Forlag.

Latner, A. L., Turner, D. M. & Way, S. A. (1966) Enzyme and Isoenzyme Studies in Preinvasive Carcinoma of the Cervix. *Lancet*, ii, 814.

Moertel, C. G. (1966) Multiple Primary Malignant Neoplasms. *Recent Results in Cancer Research*, **7**. Berlin: Springer Verlag, p. 78.